# Supplementary material for: Cytolysin A is an intracellularly induced and secreted cytotoxin of typhoidal Salmonella
Source: Nat Commun. 2024 Sep 28;15:8414. doi: 10.1038/s41467-024-52745-0 (PMC11438861; doi:10.1038/s41467-024-52745-0)

Supplementary Figures

Supplementary Figure 1

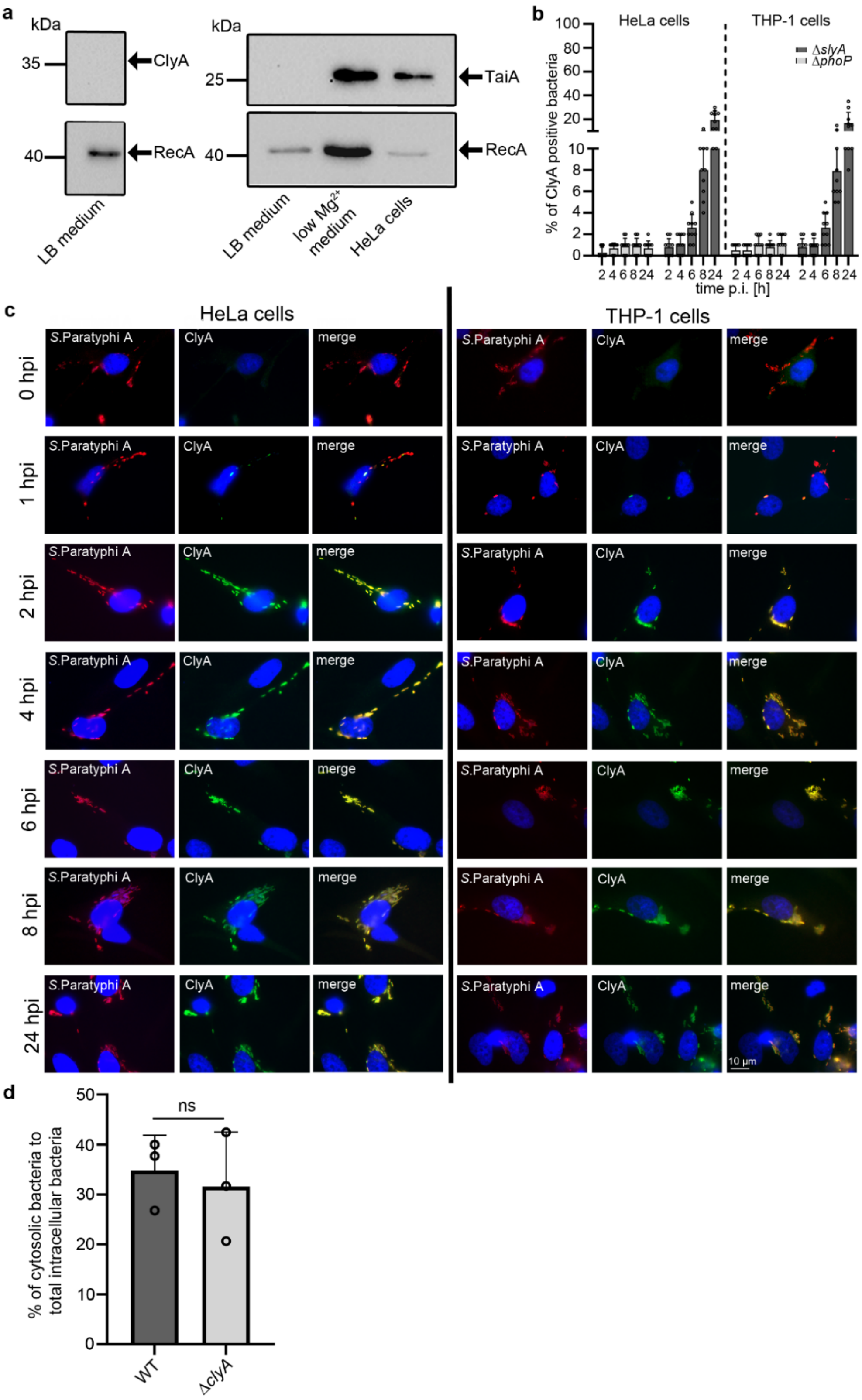

Supplementary Figure 1. (a) Western blot analysis to assess ClyA and TaiA expression after 24h growth in standard LB medium, low Mg^{2+} medium, or within infected HeLa epithelial cells. Wild-type *S.* Paratyphi strains expressing chromosomally epitope-tagged ClyA:3xFLAG (36.5 kDa) or TaiA:3xFLAG

(26 kDa) were detected using anti-FLAG antibodies. RecA (42 kDa) served as a loading control. HeLa cells were infected at MOI 100. Western blot analyses were repeated with three independent biological replicates. In **(b)**, quantification was performed on HeLa and THP-1 cells infected with Δ*phoP* or Δ*slyA* mutants expressing chromosomally epitope-tagged ClyA:3xFLAG (MOI 30). Using immunofluorescence microscopy, a total of 10 regions of interest (ROIs), each containing approximately 20 host cells (n=200), were quantitatively analyzed for each cell type. The percentage of green-positive bacteria (ClyA:3xFLAG) against the total number of red-stained bacteria (LPS) was determined and plotted. Each column represents the mean of 10 ROIs (dots). Data are presented as mean values +/- SD. In **(c)**, representative immunofluorescence microscopy images of infected HeLa and THP-1 cells (MOI 30) are shown. The cells were infected with wild-type *S*. Paratyphi A expressing chromosomally epitope-tagged ClyA:3xFLAG. At indicated time points, the cells were fixed, and antibodies were used to visualize ClyA or bacteria. **(d)** To assess the proportion of cytosolic bacteria compared to the total intracellular bacteria, a chloroquine killing assay was performed after 4 hpi as described in more detail within the methods section. Briefly, HeLa cells were infected with indicated *S*. Paratyphi A strains for 60 minutes (MOI 20). Following infection, cells were further incubated for 3 h with gentamicin. Subsequently, half of the remaining cells were treated with 400 µM chloroquine and 10 µg/ml gentamicin to kill vacuolar bacteria, while the other half was maintained in 10 µg/ml gentamicin only (representing total intracellular bacteria). After 1 hour, cells were lysed and colony-forming units (CFUs) were determined. The reported proportion represents the ratio of cytosolic bacteria to the total intracellular bacteria. Data are presented as mean values +/- SD. Statistical analysis was performed with one-way Student`s t-test. Not significant (ns): $p>0.05$. For the experiment three independent biological replicates were analyzed.

Supplementary Figure 2

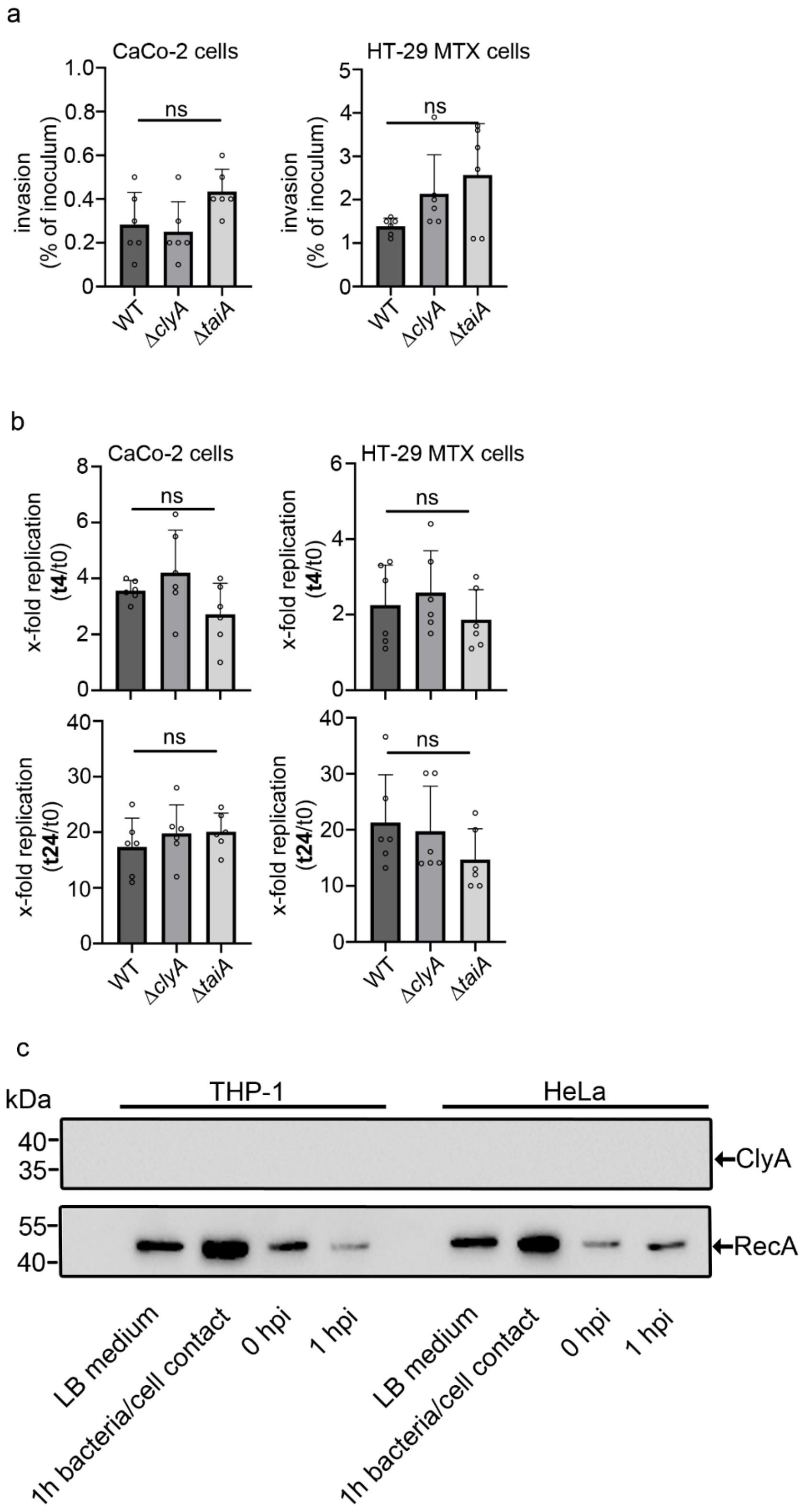

Supplementary Figure 2. (a) To determine the host cell invasion rate, Caco-2 and HT-29 MTX cells were infected with *S*. Paratyphi A wild-type, Δ*clyA*, or Δt*aiA* mutants (MOI 20). The invasion rate, expressed as a percentage of the inoculum, was determined using a gentamicin protection assay. Data are presented as mean values +/- SD. **(b)** To assess the intracellular replication rate, Caco-2 and HT-

29 MTX cells were infected with *S*. Paratyphi A wild-type, Δ*clyA* or Δ*taiA* mutants (MOI 20). The replication rate after 4 (t4) or 24 hours (t24) incubation was determined relative to the number of bacteria present at the time of uptake (0 hours). Data are presented as mean values +/- SD. For all experiments a minimum of 5 independent replicates were analyzed. Statistical analysis was performed using Ordinary one-way ANOVA and Šídák's multiple comparisons test. Not significant (ns): $p>0.05$, *: $p<0.05$, **: $p<0.01$. ***: $p<0.001$. **(c)** Western blot analysis was conducted to assess ClyA expression at various stages: before contact with THP-1 or HeLa cells (LB medium), during contact/invasion (1 hour bacteria/cell contact), intracellularly immediately after invasion (0 hours post-infection, 0 hpi), and 1 hour post-infection (1 hpi). Wild-type *S*. Paratyphi strains expressing chromosomally epitope-tagged ClyA:3xFLAG (36.5 kDa) were detected using a primary mouse anti-FLAG antibody and a secondary anti-mouse-HRP antibody. RecA (42 kDa), detected by a rabbit anti-RecA antibody, was utilized as a loading control. Western blot analyses were repeated with three independent biological replicates.

Supplementary Figure 3

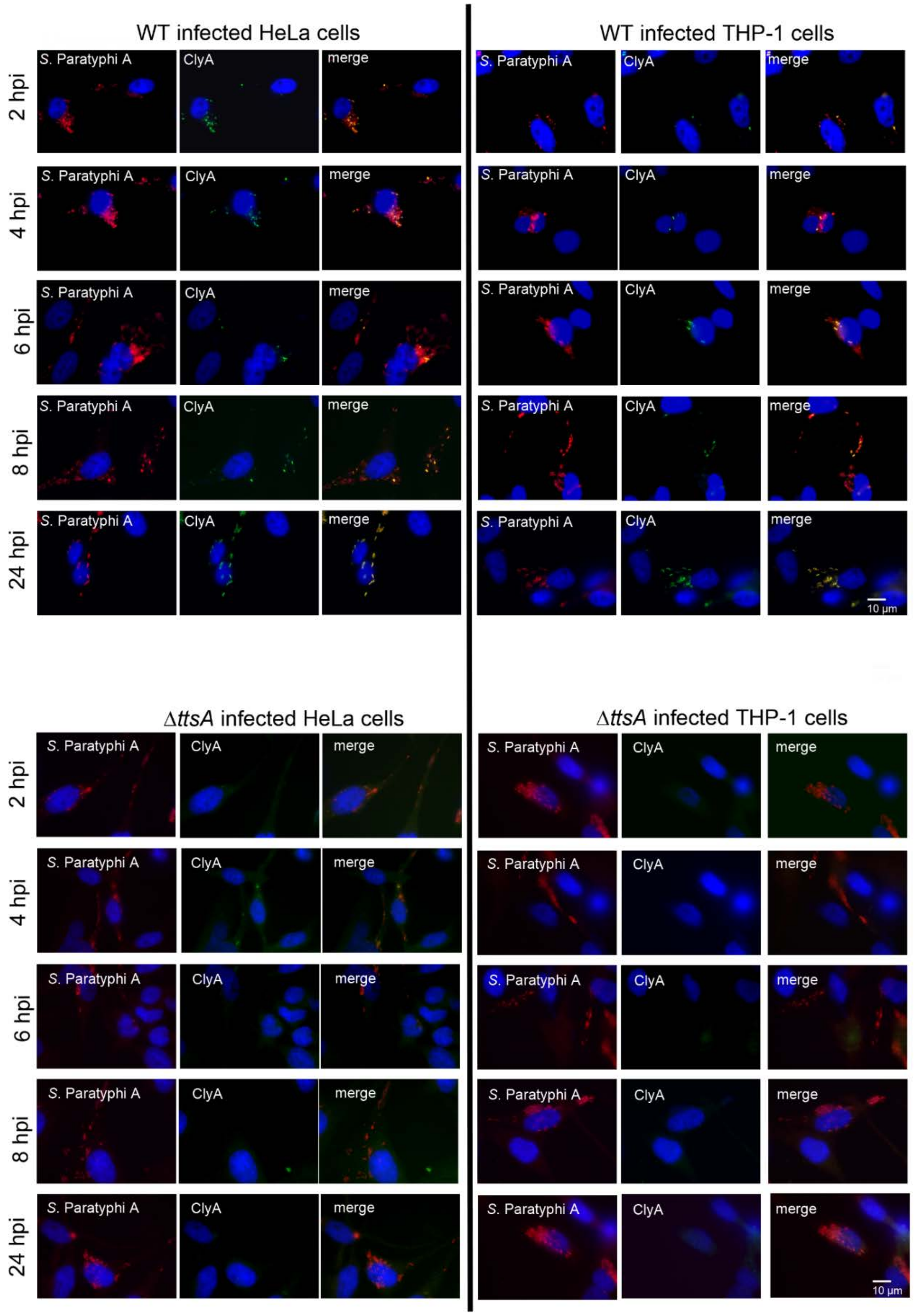

Supplementary Figure 3. Representative immunofluorescence microscopy images of infected HeLa and THP-1 cells with wild-type *S.* Paratyphi expressing chromosomally epitope tagged ClyA:3xFLAG

(MOI 30). After indicated time points, the cells were fixed and stained for *S*. Paratyphi A using a rabbit-α-LPS antibody (1:500) and for ClyA:3xFLAG using a mouse-α-FLAG antibody (1:10,000). Alexa Fluor™ 594 goat anti-rabbit and Alexa Fluor™ 488 rabbit anti mouse were used as secondary antibodies at a dilution of 1:5,000. DAPI (1:10,000, Sigma Aldrich) was used for DNA staining.

Supplementary Figure 4

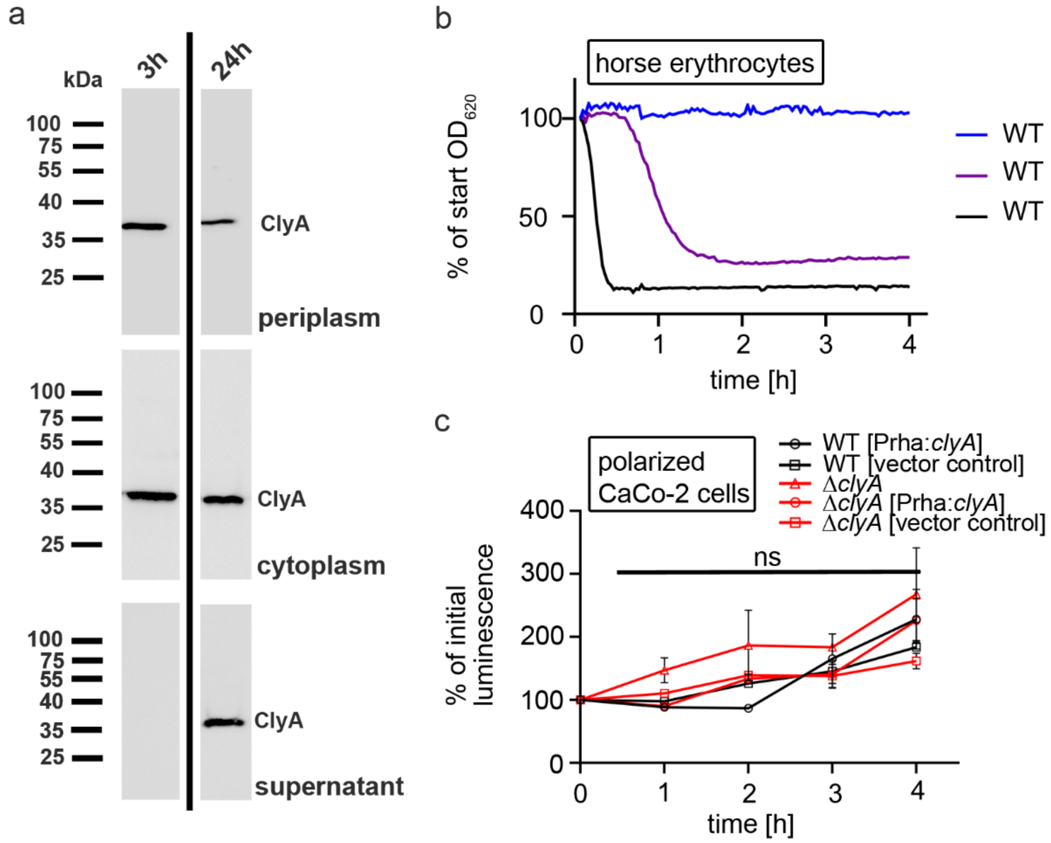

Supplementary Figure 4. (a) Western blot analyses of rhamnose induced ClyA expression in LB medium. *S*. Paratyphi A wild-type expressing *clyA:3xFLAG* on a rhamnose inducible plasmid was grown in LB with 0.1% rhamnose for indicated time points. Bacteria were harvested and subcellular fractions were isolated and collected as described in material and methods. Western blot analyses were repeated with three independent biological replicates. **(b)** Supernatants of different *S*. Paratyphi A strains (wild-type, *Prha*:*slyA* and *Prha*:*clyA*) were collected after 24 hours under plasmid- inducing conditions (LB + 0.1% rhamnose) filtered and incubated with 0.1% horse erythrocytes. Optical densities (OD_{620}) were measured every 2 minutes for up to 4 hours in a plate reader (Clario Star). **(c)** Supernatants of *S.* Paratyphi A [P*rha:clyA*], Δ*clyA* mutant, complemented Δ*clyA* mutant A [P*rha:clyA*], and empty vector controls grown for 24 hours under plasmid-inducing conditions (LB + 0.1% rhamnose), were collected and filtered (0.45 µm). Cell-free supernatants were incubated with polarized Caco-2 cells, for up to 4 hours. Samples were taken every hour and LDH-release was measured in a luminescence plate reader (Clario Star). Data are presented as mean values +/- SD. For the experiment three independent replicates were analyzed.

Supplementary Table 1. Bacterial strains and plasmids

TG0026	*S.* Paratyphi A	WT strain 45157	[56]
TG0028a	*S.* Paratyphi A	*ΔclyA*	This work
TG0031a	*S.* Paratyphi A	clyA:3xFLAG	This work
TG0033	*S.* Paratyphi A	clyA:3xFLAG<br>*slyA::aph*	This work
TG0035	*S.* Paratyphi A	clyA:3xFLAG Δ*phoP*	This work
TG0037	*S.* Paratyphi A	clyA:3xFLAG Δ*ttsA*	This work
TG0039	*S.* Paratyphi A	*Δ1307*	This work
TG0041	*S.* Paratyphi A	1307:3xFLAG	This work
TG0042	*S.* Paratyphi A	Δ1307 *ΔclyA*	This work

pTG0068	pT10 recA:3xFLAG	[64]
pTG0074	pT12 *slyA* (SPA)	This work
pTG0075	pT10 clyA:3FLAG (SPA)	This work
pTG0076	pWRG717 3xFLAG	This work
pTG0077	pT10 SPA1307-3xFLAG	This work
pTG0086	pT12 *clyA*	This work
pTG0090	pT12 SPA1307	This work
pTG0091	pT10 ompA:3xFLAG	This work
pTG0092	pT10 malE:3xFLAG	This work
pWRG730	plasmid for mutagenesis	[57]
pWRG717	plasmid for mutagenesis	[57]
pKD4	plasmid for mutagenesis	[59]
pSU312	plasmid for mutagenesis	[58]
pCP20	plasmid for mutagenesis	[59]
pWRG435	RFP-T plasmid	[67]

pTG0097	pWRG435 with *ssaG*- promoter	This work
pTG0098	pWRG435 with *uhpT*-promoter	This work
pTG0014	pT12, empty vector, rhamnose inducible promoter	[68]

Supplementary Table 2. Primer used for cloning

clyA-scarless-for	AGCTTAATTGCAATTTATATATTTAAAGAGGCAAATGATTAGGGTT TTCCCAGTCACGAC
clyA-scarless-rev	CTATCGGGCGTTAAAAGTAAACAGAGCGAATGAAAATGTATGCTT CCGGCTCGTATGTTG
clyA-cleanDel-for	TTTTGGTCGTATTCTGGAAAAAATGCAGCA
clyA-cleanDel-rev	CTATCGGGCGTTAAAAGTAAACAGAGCGAATGAAAATGTAAATCA TTTGCCTCTTTAAAT
check-SPA-clyA-del-for	CTTTTGGTCGTATTCTGGAA
check-SPA-clyA-del-rev	TGCTTCAACTTTCGATCAAA
clyA_3xF-pTG51-for	AAGGCACGGTAAGAAGACGCTTCTCGAGGTTCCTGACATCGACTA CAAAGACCATGACGG
clyA_3xF_pTG 51-rev	CTATCGGGCGTTAAAAGTAAACAGAGCGAATGAAAATGTATCCTC CTTAGTTCCTATTCC
SPA-SlyA-scarless-for	TAAAATCAGCATAATAACTTAGCAAGCTAATTATAAGGAGAGGGTT TTCCCAGTCACGAC
SPA-SlyA-scarless-rev	TTTACGTGTGGTCACATGGCCACACGTATGCCCCTGCACCTGCTT CCGGCTCGTATGTTG
SPA-slyA-cleandel-for	AGATCCATTAACAGGGGATGCCAGA
SPA-slyA-cleandel-rev	TTTACGTGTGGTCACATGGCCACACGTATGCCCCTGCACCCTCCT TATAATTAGCTTGCT
slyA control-for	TGTTCCTTTGCGTCAGAC
slyA control-rev	AAAGACGATGGCAACACC
PhoP-scarless-for	AACGCTAGACTGTTCTTATTGTTAACACAAGGGAGAAGAGAGGGT TTTCCCAGTCACGAC
PhoP-scarless-rev	CCGCAGCGACAGCGGCAGAAAATGGCGAGCAAATTTATTCTGCTT CCGGCTCGTATGTTG
PhoP-clean-Del-for	TGAAGGGCGTCAGCAAGCTGGAAGT
PhoP-clean-Del-rev	CGCAGCGACAGCGGCAGAAAATGGCGAGCAAATTTATTCACTCTT CTCCCTTGTGTTAAC
phoP control-for	GAGGGTGACTATTTGTCTGG
phoP control-rev	AAAACAGGTTGCTTTCGCC
ttsa-scarless-for	CATTCAGGTACGTATTTTATTAATATTAGAAGGAGTTATTAGGGTT TTCCCAGTCACGAC
ttsa-scarless-rev	ATGCTATCATCACAAACCGCTATAGTGGTAAGTAAGGGAATGCTT CCGGCTCGTATGTTG
ttsa-cleandel-for	AAAGGCCGTGGCGCTAGTTT

ttsa-cleandel-rev	ATGCTATCATCACAAACCGCTATAGTGGTAAGTAAGGGAAAATAACTCCTTCTAATATTA
ttsASPAseq-for	GAGACTTAGTGGCTTTCACC
ttsASPAseq-rev	TCAATCATAGCATTCAGGTACG
SPA1307 control-for	GTGAAGTGAGCGACGTTT
SPA1307 control-rev	TTCCTACCTATTGCCTCC
1307-cleandel-for	CACCGGGCGCAATCGGATCT
1307-cleandel-rev	GTTAGCATTCCAGAAGCTGGAAATACTGCCAACTTCCTGCTAACTTTACCTTTTTAATTA
scarless-SPA1307-for	GAGTGCTATTTTTTTTTGGCTAATTAAAAAGGTAAAGTTAAGGGTTTTCCCAGTCACGAC
1307-717-3xflag-rev	GTTAGCATTCCAGAAGCTGGAAATACTGCCAACTTCCTGCTGCTTCCGGCTCGTATGTTG
1307-3FLAG-TC2-for	TTTATATGGGAGTGACAAAATATAAACCCA
1307-3FLAG-TC2-rev	GTTAGCATTCCAGAAGCTGGAAATACTGCCAACTTCCTGCTTACTATTTATCGTCGTCAT
1307-717-3xflag-for	TCTGGAAAAAATGCAGCAGAGTAATCGTACCCAGCGTAGAGACTACAAAGACCATGACGG
1307-717-3xflag-rev	GTTAGCATTCCAGAAGCTGGAAATACTGCCAACTTCCTGCTGCTTCCGGCTCGTATGTTG
pt12-slyA-for	AAATTCAGGAGGAATTCACCGTGAAATTGGAATCGCCACT
pT12-slyA-rev	GCAGGTCGACTCTAGAGGATTCAATCGTGAGAGTGCAATT
SPA-clyA-3xFLAG-rev	CCGTCATGGTCTTTGTAGTCGATGTCAGGAACCTCGAGAA
SPA-pT10-clyA-for	AAATTCAGGAGGAATTCACCATGACTGGAATATTTGCAGA
pwrg717-3FLAG-for	CGATTAAGTTGGGTAACGCCGACTACAAAGACCATGACGG
pwrg717-3FLAG-rev	GTCGTGACTGGGAAAACCCTTTACTATTTATCGTCGTCAT
3xFLAG-pWRG717-v-rev	CCGTCATGGTCTTTGTAGTCGGCGTTACCCAACTTAATCG
3xFLAG-pWRG717-v-for	ATGACGACGATAAATAGTAAAGGGTTTTCCCAGTCACGAC
pT10_SPA1307-for	TAATGAAATTCAGGAGGAATTCACCATGAAACTATCCGTAACATT
pT10_SPA1307-rev	AATCACCGTCATGGTCTTTGTAGTCTCTACGCTGGGTACGATTAC
pT12_clyA-rev	GCAGGTCGACTCTAGAGGATTCAGATGTCAGGAACCTCGA
SPA-pT10-clyA-for	AAATTCAGGAGGAATTCACCATGACTGGAATATTTGCAGA
pT12-SPA1307-rev	GCAGGTCGACTCTAGAGGATTTATCTACGCTGGGTACGAT
pT10_SPA1307-for	TAATGAAATTCAGGAGGAATTCACCATGAAACTATCCGTAACATT
pT10-ompA-rev	CCGTCATGGTCTTTGTAGTCAGCCTGCGGCTGAGTTACCA
pT10-ompA-for	AAATTCAGGAGGAATTCACCATGAAAAAGACAGCTATCGC
pT10-malE-rev	CCGTCATGGTCTTTGTAGTCCTTGGTGATACGGCTTTGTG
pT10-malE-for	AAATTCAGGAGGAATTCACCATGAAGATTAAAACTGGCGT

EcoRI-3xFLAG-for	CTAAGAATTCGACTACAAAGACCATGACGGTGATTATAAAGATCATGACATCGACTACAAAGATGACGACGATAAATAGTAA
EcoRI-3xFLAG-rev	TTACGAATTCCATATGAATATCCTCCTTAG
3xFLAG-long-for	GACTACAAAGACCATGACGGTGATTATAAAGATCA
pTG51 check-rev	ACTTTGCAGGGCTTCCCA
pTG51 check-rev	ACTTTGCAGGGCTTCCCA
pTG51seq-for	CATTTCCCCGAAAAGTGC
pTG51seq-rev	GTTCGCTTGCTGTCCATA
pTG51 check-rev	ACTTTGCAGGGCTTCCCA
pWRG435-rev	TACCGAGCTCGAATTCTTGA
pWRG435-for	CGGGTATCGCTGAAAATGTT
pwrg435_ssaG-P-for	TCAAGAATTCGAGCTCGGTACACATCCGGAAACCAATATG
pwrg435_ssaG-P-rev	AACATTTTCAGCGATACCCGAATGCTTTTCCTTAAAATAA
pwrg435_uhpT-P-for	TCAAGAATTCGAGCTCGGTACGCAAAGAAATCCTCGCTAA
pwrg435_uhpT-P-rev	AACATTTTCAGCGATACCCGGGATTACTCCTGAGCTAATT

References (only cited in the Supplementary Information):

67. Bender JK, Wille T, Blank K, Lange A, Gerlach RG. LPS structure and PhoQ activity are important for Salmonella Typhimurium virulence in the Galleria mellonella infection model [corrected]. PLoS One. 2013;8(8):e73287. Epub 2013/08/21. doi: 10.1371/journal.pone.0073287. PubMed PMID: 23951347; PubMed Central PMCID: PMCPMC3738532.
68. Dietsche T, Tesfazgi Mebrhatu M, Brunner MJ, Abrusci P, Yan J, Franz-Wachtel M, et al. Structural and Functional Characterization of the Bacterial Type III Secretion Export Apparatus. PLoS Pathog. 2016;12(12):e1006071. Epub 20161215. doi: 10.1371/journal.ppat.1006071. PubMed PMID: 27977800; PubMed Central PMCID: PMCPMC5158082.

Uncropped supplementary Western Blots:

Figure 1a and suppl Figure 1a anti Flag

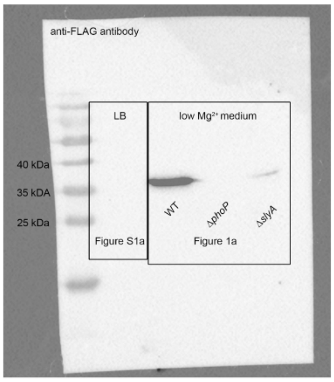

suppl Figure 1b anti Flag

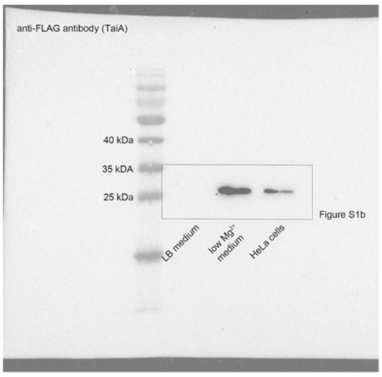

Figure 1a and suppl Figure 1a anti recA

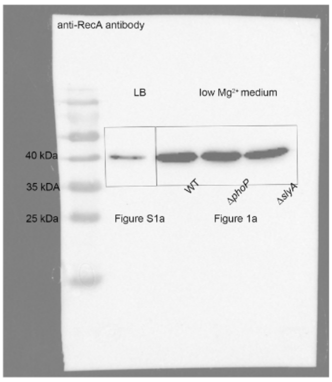

suppl Figure 1b anti recA

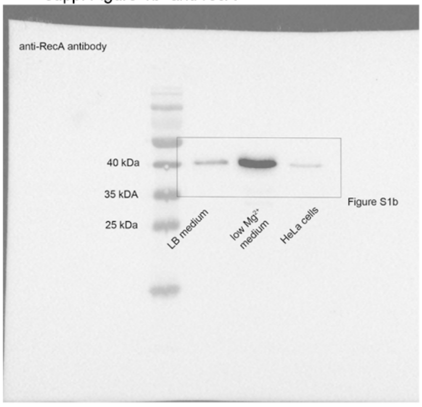

suppl Figure 2c anti RecA

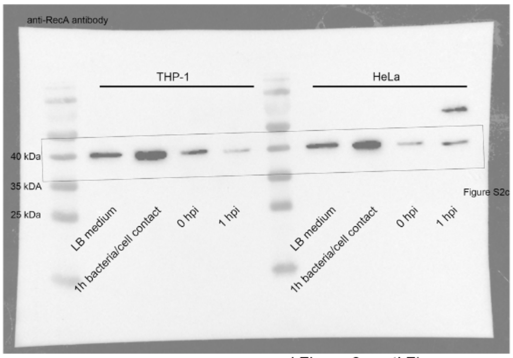

suppl Figure 2c anti Flag

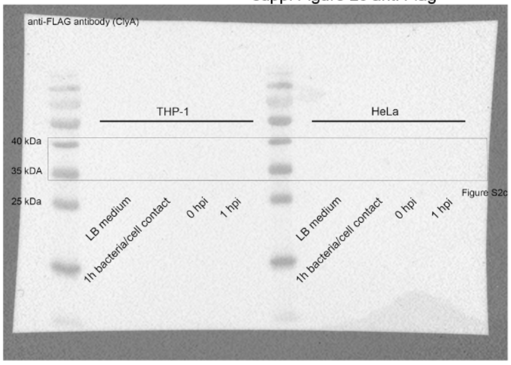

Supplement: Supplementary file 1 — Supplementary Information [file 41467_2024_52745_MOESM1_ESM.pdf]
