# Supplementary figures and images for: Cytolysin A is an intracellularly induced and secreted cytotoxin of typhoidal Salmonella
Source: Nat Commun. 2024 Sep 28;15:8414. doi: 10.1038/s41467-024-52745-0 (PMC11438861; doi:10.1038/s41467-024-52745-0)

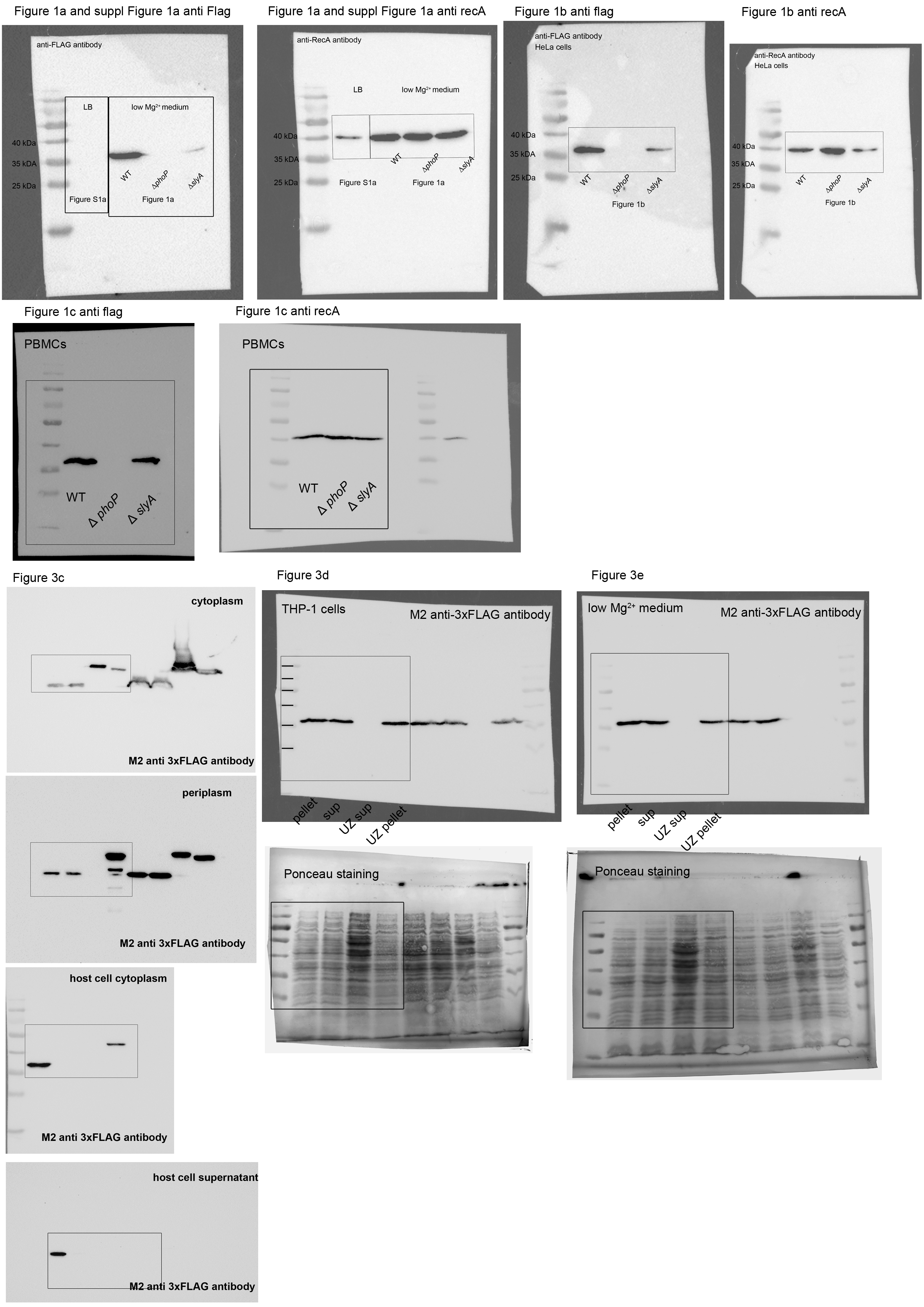

Supplement: Supplementary file 4 — Source Data [file 41467_2024_52745_MOESM4_ESM.xlsx › Source Data files Blots.tif]
